# Effect of accelerated aging on the thermo-mechanical behavior and biotribological properties of an irradiation cross-linked GO/UHMWPE nanocomposite after VE diffusion

**DOI:** 10.1039/d4ra05720a

**Published:** 2024-10-11

**Authors:** Yinbiao Li, Weipeng Duan

**Affiliations:** a School of Mechanical Engineering, Wuxi Institute of Technology Wuxi 214121 China weipengduan@126.com

## Abstract

In this work, the influence of accelerated aging on the thermo-mechanical behavior and biotribological properties of an irradiation cross-linked GO/UHMWPE nanocomposite after VE diffusion was investigated, including through differential scanning calorimetry (DSC), gel content, FT-IR characterization, oxidation index, ball indentation hardness, and especially the biotribological properties. The results show that accelerated aging increased the melting point and crystallinity of the nanocomposite, but resulted in a decrease in thermal stability and gel content. The oxidation index increased by 60.2% and the hardness decreased by 18.1%. In particular, the friction coefficient and wear rate increased by 99.5% and 87.4% respectively. A simple VE diffusion process had no obvious effect on the melting point, crystallinity, thermal stability, gel content and hardness, but the oxidation resistance and biotribological performance were improved to a certain extent. On the contrary, when VE exists in the accelerated aging process, the above properties are significantly improved. In particular, the oxidation index decreased by 21.1%, and the friction coefficient and wear rate decreased by 33.7% and 26.4%, respectively. After accelerated aging, fatigue wear and abrasive wear are the main wear forms, while VE plays the function of reducing friction and wear. Besides, the anti-friction and wear resistance mechanism of VE during the accelerated aging process was also illustrated.

## Abstract 2

The influence of accelerated aging on the thermo-mechanical behavior and biotribological properties of irradiation cross-linked GO/UHMWPE nanocomposite after VE diffusion was investigated, especially the biotribological properties.

## Graphical abstract

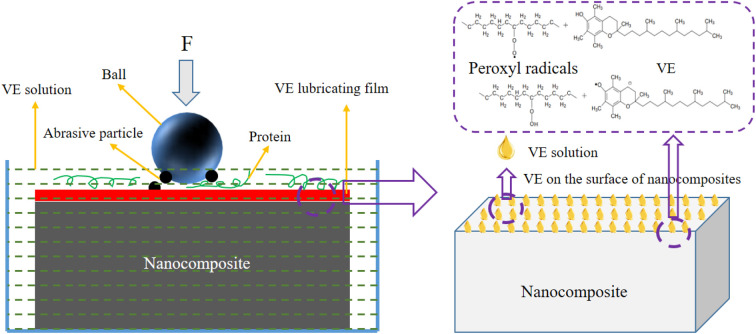

## 1. Introduction

In recent years, much more attention has been paid to the application of UHMWPE in the artificial joint field.^1–3^ More remarkably, due to its excellent mechanical and tribological properties, graphene oxide/ultrahigh molecular weight polyethylene (GO/UHMWPE) and its irradiation cross-linked nanocomposite has been widely investigated in the field of artificial joint replacement.^4–6^ However, the oxidation and wear of UHMWPE implanted in the human body will affect its performance.^7,8^ As a clinical problem, osteolysis caused by these has always been one of the important factors limiting the long-term service life.^9,10^ Vitamin E (VE), a natural antioxidant that can absorb and stabilize free radicals, has also attracted great attention in the modification of UHMWPE. As an effective technique of enhanced modification, adding VE can be used in many aspects in the coming future.^11,12^

Up to now, the literature shows that there are two methods of VE modifying UHMWPE and its nanocomposites: blend and diffusion. Terkawi *et al.* studied the performance of a VE-blend UHMWPE material for prosthetic components, and proved a better mechanical performance with lesser adverse cellular responses when compared with conventional polyethylene in experimental animal models.^13^ Pang *et al.* reported a VE-blend irradiation cross-linked GO/UHMWPE nanocomposite, and found compared with UHMWPE, the micro-hardness, young's modulus, yield stress and fracture stress improved by 8%, 28%, 33% and 37% respectively, whereas, the oxidation index also increased to 1.7.^14^ Saikko *et al.* made thin, large-diameter acetabular liners by VE-blend highly cross-linked UHMWPE and studied its wear and friction property in calf serum. The results indicate that the composites showed reasonable wear and friction behavior, the mean wear rate was 1.9 times lower than conventional total hip prostheses.^15^ Sagbas *et al.* used VE-blended UHMWPE to prepare acetabular prosthesis and paired it with CoCrMo femoral head to study the tribological properties. The results show that this method can significantly reduce the heat generated by friction and reduce the harmful effects on the surrounding tissues and lubricants around artificial joints.^16^ Su *et al.* compared the wear rate of UHMWPE, highly cross-linked UHMWPE and VE-blend highly cross-linked UHMWPE using a hip joint simulator and found that when using the same femoral head material, the wear rate of highly cross-linked UHMWPE and VE-blend highly cross-linked UHMWPE liner was lower than that of UHMWPE liner, and the wear rate of highly cross-linked UHMWPE liner increased after blend with VE.^17^ It can be seen that VE can significantly improve the oxidation stability and partial mechanical properties of irradiation cross-linked UHMWPE artificial joints. However, it is a tricky problem that VE hinders cross-linking and endangers the wear of UHMWPE.^18–20^

VE diffusion, as another modification method, has also received extensive attention. Since VE does not exist during irradiation, it will not hinder cross-linking. Oral *et al.* studied the diffusion of VE into high cross-linked UHMWPE and found that the cross-linking degree did not change significantly. The accelerated aging test showed that the material had excellent oxidation resistance, and the impact strength and ultimate tensile strength greatly improved.^21,22^ Wolf *et al.* Studied the diffusion of VE in irradiation cross-linked UHMWPE under different diffusion conditions (conventional conditions and supercritical CO_2_ conditions). The results show that the diffusion depth and mechanical properties can obtain better results under supercritical CO_2_ conditions.^23^ Salemyr *et al.* compared the VE diffused high cross-linked UHMWPE liner with the standard joint liner, and found that after years of clinical research, the former exhibits excellent performances.^24^ Nebergall *et al.* conducted a follow-up study on the VE diffused irradiated cross-linked UHMWPE material for up to 5 years. The results show that the joint implant still has excellent performance after 5 years of use.^25^ In our previous work, VE diffused irradiation cross-linked GO/UHMWPE composite was prepared and the thermal stability and mechanical property was tested, the results show that oxidation stability of the nanomaterial is improved, and this method can be used to modify artificial joint nanocomposites.^26^

Based on the above analysis, it can be found that there has been some research on VE diffusion modified UHMWPE nanocomposites. In addition, aging is also one of the important factors affecting its life in long-term service. In this respect, Huang *et al.* studied the effect of accelerated aging on GO/UHMWPE nanocomposites, the results show that accelerated aging can slightly increase the melting temperature of nanocomposite, but significantly improve crystallinity and wetting properties. However, after accelerated aging, the cross-linking density of GO/UHMWPE significantly decreased.^27^ On this basis, Huang *et al.* evaluated the mechanical properties of nanocomposites after accelerated aging. The results indicate that after accelerated aging, the mechanical properties and crack resistance significantly reduced.^28^ Recently, Huang *et al.* investigated the effect of aging on the tribological performance of GO/UHMWPE in water lubrication and found aging caused a decrease of 14.74% in compressive strength and 21.12% in yield stress of composite materials, respectively. In addition, the aging process also increases the friction coefficient, leading to a decrease in wear resistance.^29^

However, few reports on the effect of accelerated aging on its biotribological properties of irradiation cross-linked GO/UHMWPE nanocomposite after VE diffusion. According to this, GO/UHMWPE nanocomposites were prepared first and then irradiated by γ-rays, following with VE diffusion and accelerated aging. Differential scanning calorimeter (DSC), gel content, FT-IR characterization, oxidation index, ball indentation hardness and biotribological property in calf serum, were investigated. This work aims to explore the influence of accelerated aging on the biotribological properties of irradiation cross-linked GO/UHMWPE nanocomposites after VE diffusion. Besides that, the anti-friction and wear resistance mechanism was also illustrated.

## 2. Materials and experimental

### 2.1 Materials

Medical UHMWPE powder, with an average particle size of 140 μm and mean molecular weight of 5 million, was provided by Ticona Company, USA. High-purity graphite powder, 99.9% purity and 325 mesh, was offered by Qingdao Jinrilai Co., Ltd, China. Medical grade VE solution, 99.9% purity, was purchased from Pharmaceutical Group Co., Ltd, China. Calf serum was provided by Zhejiang Tianhang Biotechnology Co., Ltd, China. Other chemical reagents, such as absolute ethanol and deionized water, were of analytical grade and also come from purchased from Pharmaceutical Group Co., Ltd, China.

### 2.2 Materials preparation

GO was prepared on the basis of modified Hummers' method.^30^ 0.5 wt% GO/UHMWPE nanocomposite was made based on its excellent mechanical properties that investigated in previous reports,^31,32^ the steps are as follows. First, 0.25 g GO and 49.75 g UHMWPE powder was put in a beak with 100 ml absolute ethanol, and ultrasonic dispersion for 1 h by ultrasonic cleaning machine (BG-06C, Guangzhou Bangjie Electronic Products Co., Ltd, China). Second, the mixed solution was ball milled at a speed of 400 rpm for 2 h in the planetary ball mill (QM-3SP2, Nanjing Nanda Instrument Co., Ltd, China). Third, remove absolute alcohol from the mixed solution in a water bath (LKTC-B1-T, Jintan Liangyou Instrument Co., Ltd, China) at 60 °C until it totally dried. Fourth, mixed powder was placed in home-made mold with a size of 40 mm × 40 mm × 5 mm and pressed on a plate vulcanization machine (XLB 400, Qingdao Xincheng Yiming Rubber Co., Ltd, China) at a pressure of 5 MPa for 0.5 h, and put it in a vacuum drying oven (DZF-6020, Shanghai Precision Instrument Co., Ltd) at 200 °C for 2 h. Fifth, the melted powder was pressed under a pressure of 10 MPa until cooled to room temperature and demolded to obtain GO/UHMWPE nanocomposite. Sixth, the nanocomposite was irradiated by γ-rays (AB2.5-40, Nanjing Xiyue Technology Co., Ltd, China) at an irradiation rate of 0.5 kGy h^−1^, and the total irradiation dose was 100 kGy. Last but one, the irradiation cross-linked GO/UHMWPE nanocomposites was immersed in a beak with 500 ml VE solution at 120 °C for 48 h, then taken it out and homogenized in a drying oven at 120 °C for 48 h, and cooled to room temperature. Last, parts of the nanocomposites were placed in an electric thermostatic drying oven at 80 °C for 21 days for accelerated aging treatment, which provided by Shanghai Boxun Industry & Commerce Co., Ltd.^26^ The lubricating fluid was 25% calf serum solution composed of calf serum and an appropriate amount of deionized water. It is worth noting that in this work, irradiation cross-linked GO/UHMWPE nanocomposite was recorded as I-GO/UHMWPE, the material diffused by VE was called VE+I-GO/UHMWPE, those two undergo accelerated aging treatment were called Aged+I-GO/UHMWPE and Aged+VE+I-GO/UHMWPE. In addition, all the experiments were repeated 5 times, and the average value was taken as the data in this work.

### 2.3 Differential scanning calorimeter (DSC)

Differential scanning calorimeter (DSC Q2000, TA Instruments, USA) was used to analyze the melting temperature and crystallinity of the samples in the nitrogen atmosphere, at a heating rate of 10 °C min^−1^ from 20 to 180 °C. The samples were first heated to 180 °C and held for 5 min then cooled to 20 °C at a cooling rate of 10 °C min^−1^. Crystallinity was calculated by following formula (1).^20^1
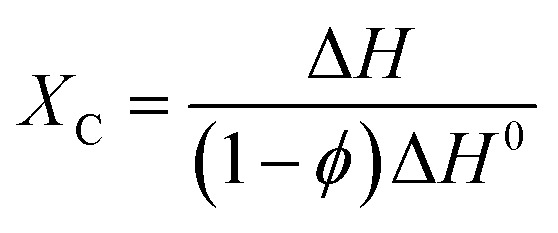
where, *X*_C_ is crystallinity, Δ*H* is the melting enthalpy, *ϕ* is the content of GO, Δ*H*^0^ is the melting enthalpy of 100% crystalline UHMWPE, and the data is 293 J g^−1^.

### 2.4 Thermogravimetric analysis (TGA)

In order to study the thermal stability of the nanocomposites, thermogravimetric analysis (TGA) was also investigated by a thermogravimetric analyzer (TGA/1, Switzerland), which temperature ranges from 50 to 800 °C at a rate of 20 °C min^−1^ in nitrogen atmosphere.

### 2.5 Gel content

Gel content was tested according to ASTM D2765 and calculated by the following formula (2).^26,33^2
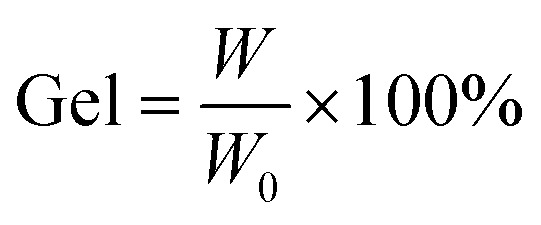
where, *W* and *W*_0_ is the quality of sample before and after test, respectively.

### 2.6 FT-IR characterization and oxidation index

Fourier transform infrared spectrometry (FT-IR, Nicolet iS10, USA) was used to character the changes of molecular structure of nanocomposites. After that, oxidation index (OI) was tested to investigate the oxidation stability of nanocomposites. On the basis standard of ASTM F2003, OI was calculated by formula (3).^26,33^3
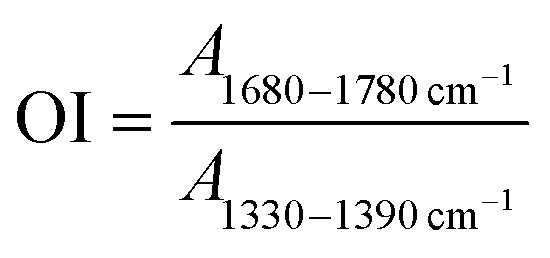
where, *A*_1680−1780 cm^−1^_ and *A*_1330−1390 cm^−1^_ represent the area enclosed by the infrared spectrum and the coordinate axis in this interval.

### 2.7 Ball indentation hardness experiment

Ball indentation hardness was tested by MFT-5000 tribometer, RTEC, USA, with a *φ* 5 mm Si_3_N_4_ ball, according to the ISO 2039-73 standard. Pressing the sample with a force of 9.8 N and keep it for 15 s, then increase the force to 132 N and keep it for 30 s, recording the indentation depth and calculated by the following formula (4).^20^4
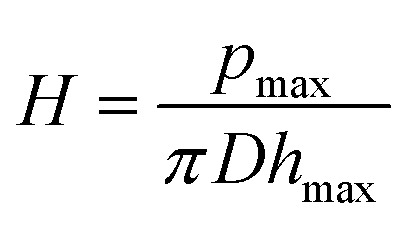
where, *H* is the indentation hardness (N mm^−2^), *D* is the diameter of the ball (mm), *p*_max_ is the maximum experimental force (N), *h*_max_ is maximum indentation depth (mm).

### 2.8 Biotribological property test

The biotribological performance of the nanocomposites were measured on a reciprocating tribometer (MFT-5000, RTEC, USA) sliding against Si_3_N_4_ ball with a diameter of 6 mm in 25% calf serum solution. The experiment was carried out with a sliding distance of 10 mm under a load force of 15 N and a frequency of 1 Hz for 1800 s.^29^ The friction coefficient (COF) and wear volume was recorded by software automatically, worn surface morphology was analyzed by scanning electron microscope (SEM, Carl Zeiss, Sigma 300, Germany). Wear rate (WR) was calculated according to the following formula (5).^34^5
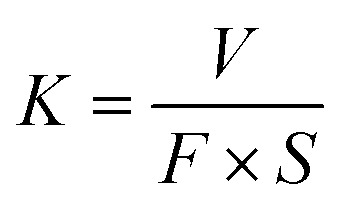
where, *K* is wear rate, *V* is wear volume, *F* and *S* represents load force and sliding distance, respectively.

## 3. Results and discussion

### 3.1 Melting temperature and crystallinity

**Fig. 1:**
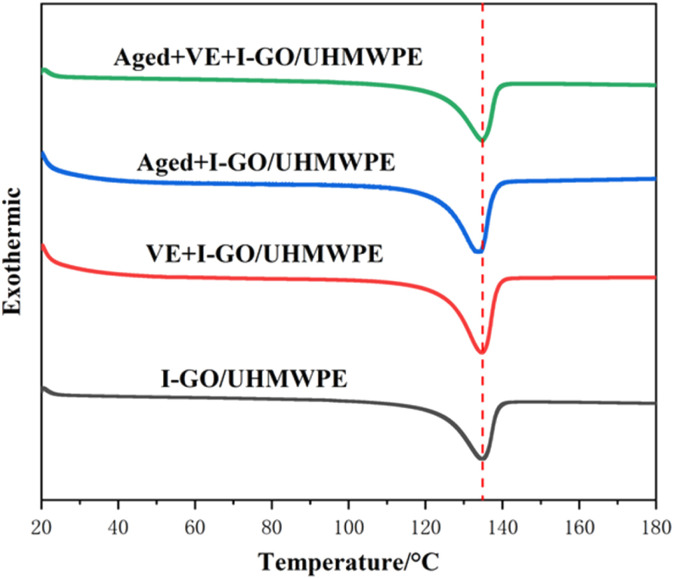
DSC curves of nanocomposites.

In order to study the thermal stability of the nanocomposites after VE soaking and accelerated aging, the melting point and crystallinity were studied by DSC, as shown in Fig. 1.

It can be seen that the melting point of I-GO/UHMWPE is 135.9 ± 0.05 °C, and the melting point turns into 136.3 ± 0.04 °C after accelerated aging. After VE soaking, the melting point of the nanocomposite is 135.7 ± 0.06 °C, and changes to 136.1 ± 0.08 °C after accelerated aging treatment. The results showed that VE soaking and accelerated aging had no obvious effect on the melting point of the nanocomposites. However, the crystallinity changed significantly. The crystallinity of I-GO/UHMWPE is 59.8%, and increased by 12.7% after accelerated aging. In the aging process, due to a long time of air atmosphere, oxygen diffuses into the nanocomposite and reacts with free radicals to produce a large number of oxides. In addition, when oxides are produced, a large number of molecular chains are broken, resulting in the decrease of molecular weight. The broken molecular chains are recrystallized in the amorphous region, which increase the crystallinity. VE immersion had no significant effect on the crystallinity, but after accelerated aging treatment, the crystallinity of the nanocomposite soaked in VE solution decreased by 5.3%. This is because, the function of absorbing and stabilizing free radicals of VE, the presence of VE reduces the interaction of free radicals. Furthermore, oily VE may acts as a protective film on the surface of the nanocomposite, reducing the entry of oxygen and avoiding the generation of oxides.^21,22^

### 3.2 Thermogravimetric analysis

**Fig. 2:**
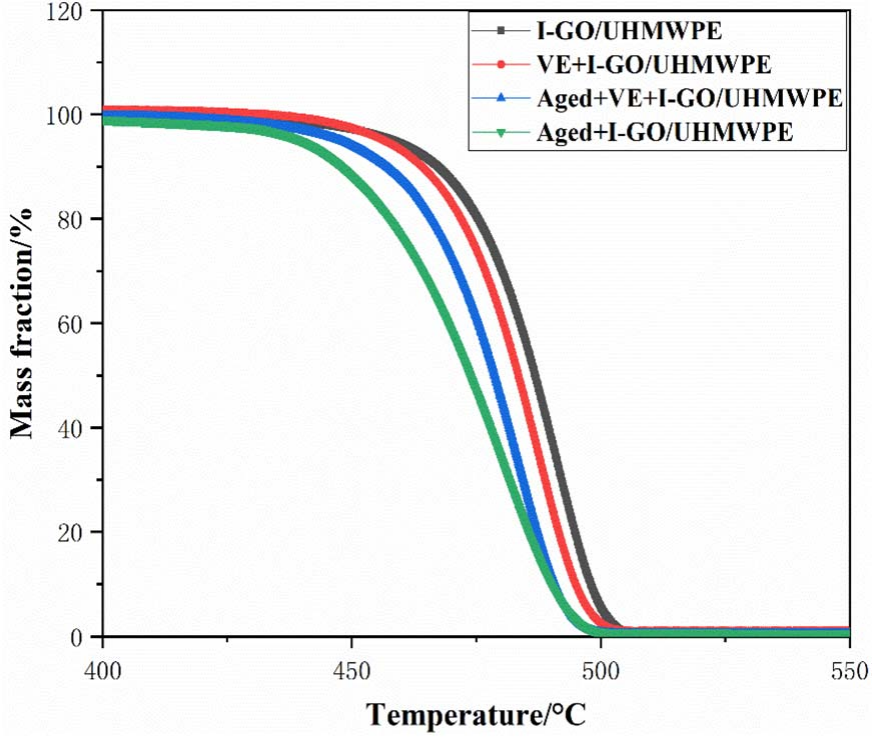
TGA curves of the nanocomposites.

As an important index to characterize thermal properties, the variation of TGA was also studied, as shown in Fig. 2. The TGA curve of I-GO/UHMWPE nanocomposite indicated that it has good thermal stability, after VE soaking, the thermal stability decreased slightly, but not obvious. Accelerated aging reduces the thermal stability. Since the composites are in high-temperature oxygen environment for a long time, high temperature promotes the fracture of molecular chains and the movement of free radicals. The reaction between free radicals and oxygen causes oxidative degradation and reduces the thermal stability. On the contrary, the existence of VE can stabilize and absorb free radicals, which reduces the degree of oxidative degradation reaction. Therefore, its thermal stability improved after accelerated aging.

### 3.3 Gel content

**Fig. 3:**
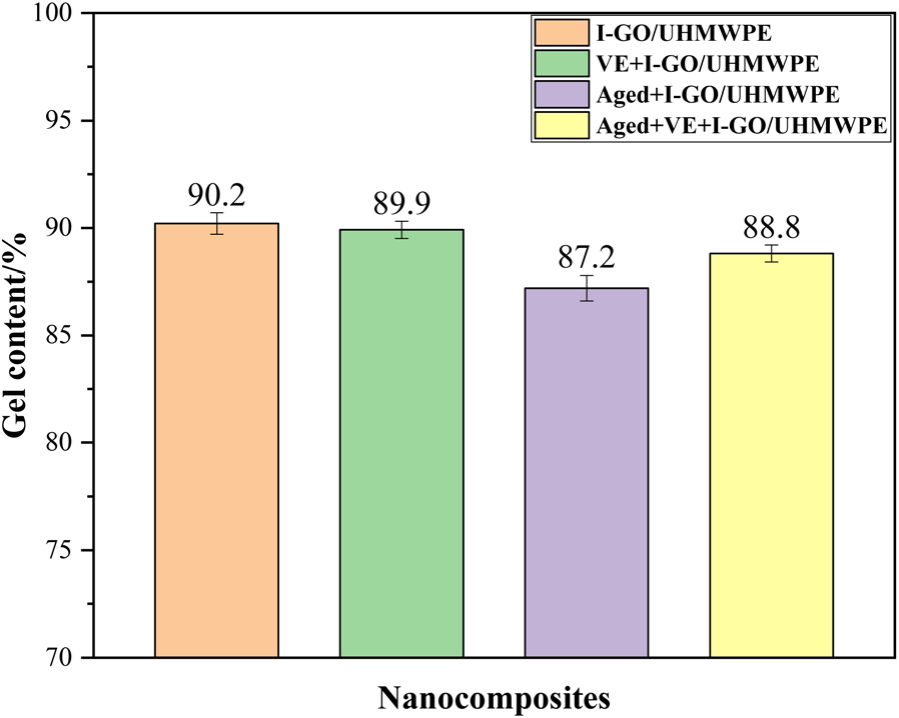
Gel content of the nanocomposites.

The gel content of VE diffused and accelerated aging nanocomposites was shown as Fig. 3. The gel content of I-GO/UHMWPE is 90.2 ± 0.5%, after VE soaking, it stays basic stability (89.9 ± 0.4%), indicating that the absence of VE during irradiation did not affect the gel content of the nanocomposites, which is consistent with the results of Pang.^14,28^ The gel content of the nanocomposite without VE decreased by 3.3% after accelerated aging (87.2 ± 0.6%), while increased by 1.8% with VE soaked first and then accelerated aging (88.8 ± 0.4%). During irradiation, a large number of free radicals were generated, such as UHMWPE and GO free radicals,^30,35^ and the cross-linking of free radicals leading to the existence of gel content. At this time, there are still some unreacted residual free radicals. On the one hand, the reaction of free radicals with O_2_ will reduce the content of free radicals, however, the large two-dimensional surface of GO limiting the diffusion of O_2_ into nanocomposite, which reduce the number of free radical reactions to a certain extent. On the other hand, high temperature accelerates the movement of free radicals, as well as GO. Although GO can hinder the diffusion of O_2_, it also hinders the cross-linking of free radicals. The combined action reduced the gel content of the nanocomposite after accelerated aging. For the nanocomposites soaked in VE first and then accelerated aging, VE can absorb some free radicals, indeed, it also plays a role in isolating oxygen diffusion, resulting in an increase of cross linked free radicals and slowed down the decrease of gel content to some extent. The decrease of gel content after accelerated aging is mainly due to the break of molecular chain and the reaction of newly generated free radicals with oxygen to produce oxidation products. In this scenario, the soaked VE not only restricts the entry of oxygen but also hinders the chain reaction of free radicals, thereby retarding the aging of irradiated UHMWPE and preserving the gel content at a relatively high level. In addition, VE can reduce the entry of oxygen and block chain reactions. The changes of gel content of composites are caused by the interaction of many aspects.

### 3.4 FT-IR characterization and oxidation index

**Fig. 4:**
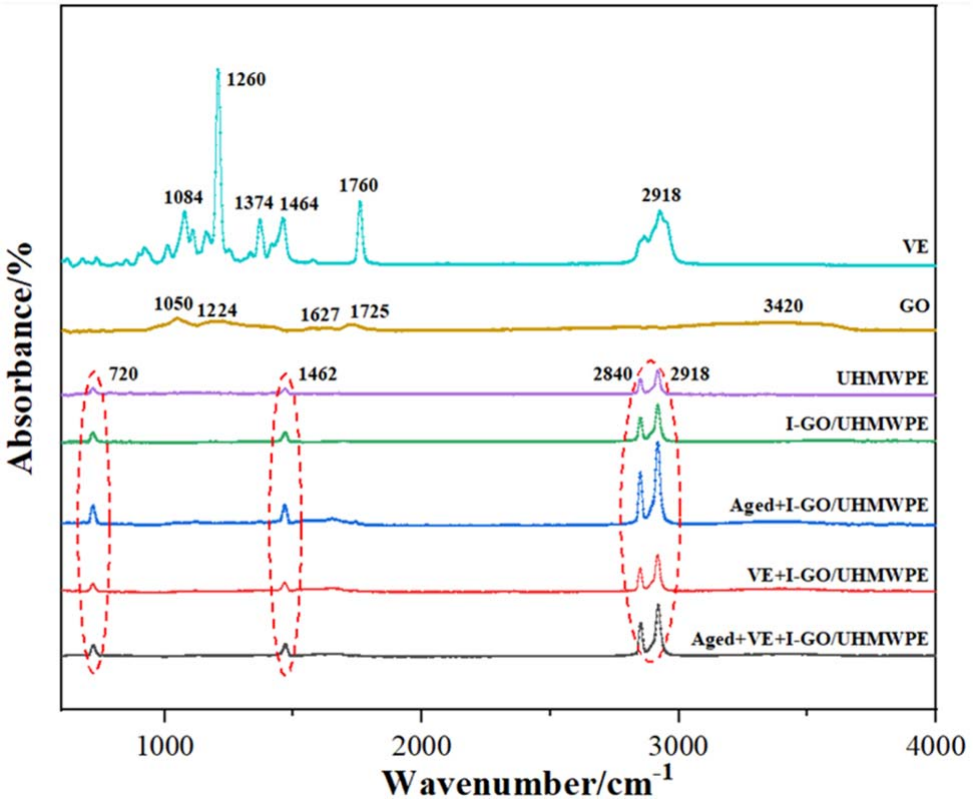
FT-IR of the nanocomposites.

**Fig. 5:**
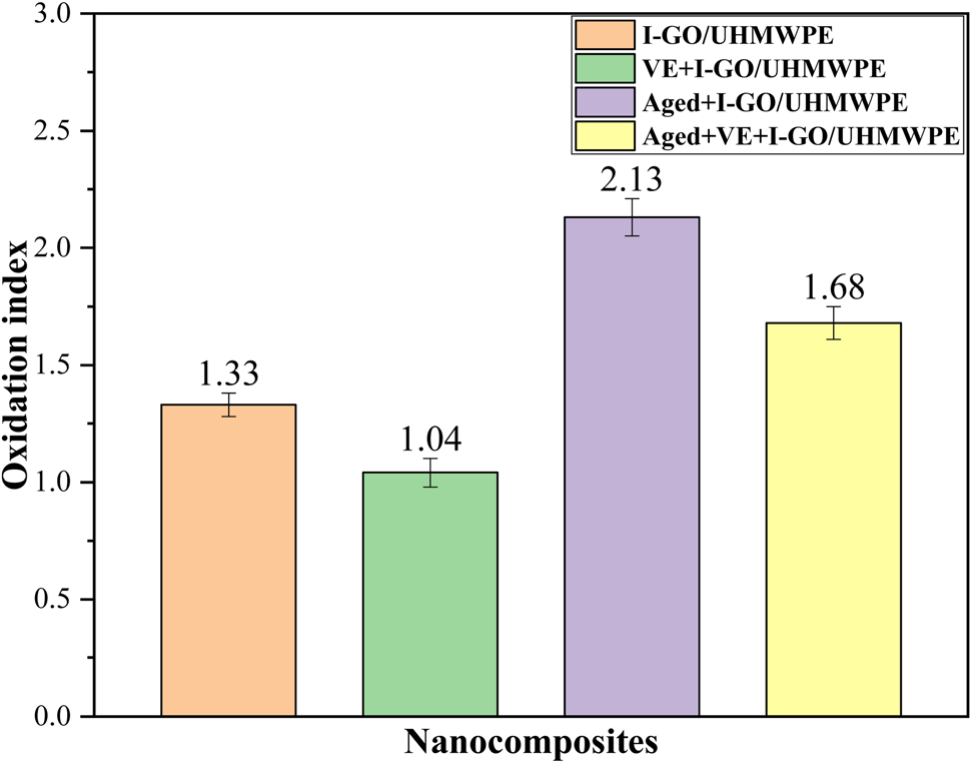
Oxidation index of the nanocomposites.

FT-IR of the nanocomposite was shown in Fig. 4. It can be seen in UHMWPE spectrum, the peaks located at 720 cm^−1^, 1462 cm^−1^, 2918 cm^−1^ and 2840 cm^−1^ was the CH_2_ rock, the bending peak of C–H bonds and the stretching absorption peaks of C–H, respectively. The C–C vibration signal at 1050 cm^−1^, the sp^2^ C–C bond and C=O stretching vibration signal at 1627 cm^−1^ and 1725 cm^−1^, the O–H stretching vibration signal at 3420 cm^−1^ was emerged in the spectrum of GO. From VE spectrum, ether group, C–O stretching of the phenol, symmetric bending, methyl asymmetric peak and stretching absorption peak of C–H lies at 1084 cm^−1^, 1260 cm^−1^, 1374 cm^−1^, 1464 cm^−1^ and 2918 cm^−1^, respectively. According to our previous researches, FT-IR results showed that there was no significant different whether GO filling or irradiation cross-linking, which suggested that the molecular structures of UHMWPE has not been significantly affected.^22,23^ Simultaneously, it can be seen that there is typical characteristic peak (1260 cm^−1^) of VE in the spectrum of different nanocomposites, indicating that VE has been diffused into the matrix of composites.^19,20^ Furthermore, it also can be seen that the FT-IR spectrum of I-GO/UHMWPE before/after VE soaking and accelerated aging is same as that of UHMWPE, and no obvious change. It is mainly due to the too small amount of VE diffusion on the surface and it further diffuses into the nanocomposite after accelerated aging, causing it too little to be detected. Above results show that VE immersion and accelerated aging treatment have no obvious effect on the molecular structure of the nanocomposites, however, the oxidation index (OI) changed significantly, as shown in Fig. 5.

It can be seen that the OI of I-GO/UHMWPE nanocomposites is 1.33 ± 0.05, and increases to 2.13 ± 0.09 after accelerated aging treatment. This is due to the oxidative degradation of the nanocomposite in high temperature air over a long period of time. After VE soaking, the OI is 1.04 ± 0.07, and it increased to 1.68 ± 0.08 after accelerated aging treatment. Compared with the nanocomposite without soaked in VE solution, the OI decreased by 21.1%. This is because VE plays the role of isolating oxygen and stabilizing free radicals, which fully shows the good antioxidant performance of VE.

### 3.5 Ball indentation hardness

**Fig. 6:**
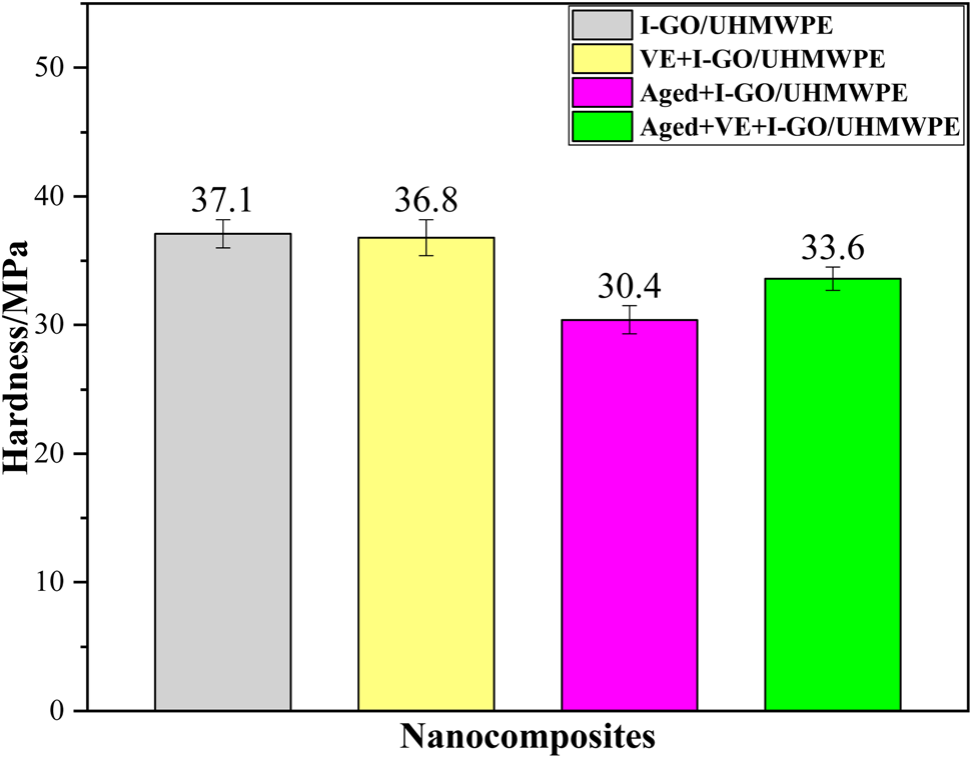
Ball indentation hardness of the nanocomposites.

The ball indentation hardness of nanocomposites was shown as Fig. 6. The ball indentation hardness of I-GO/UHMWPE is 37.1 ± 1.1 MPa. After VE soaking, the hardness remains basically unchanged (36.8 ± 1.4 MPa). A small amount of VE solution hardly significantly affects the crystallinity and gel content of the nanocomposite, therefore, no significant differences of hardness were found after VE immersion. Duan and Buchanan conducted a similar study and found that the ball indentation hardness increased with improved crystallinity of the nanocomposite.^26,35^ However, when accelerated aging is finished, the hardness of the nanocomposites decreased by 18.1% (30.4 ± 1.3 MPa). As mentioned above, although accelerated aging increases the crystallinity of the nanocomposite, it also reduces the gel content. The reasons for the decrease of hardness can be attributed to the following aspects. On the one hand, due to oxidative degradation in the aging process, the adhesion between GO and UHMWPE decreases, the capacity of GO to transfer load goes down, resulting in the decrease of hardness. On the other hand, higher temperature promotes the movement of GO in the nanocomposite, which hinders the cross-linking reaction of free radicals and reduces the gel content, thus affects the hardness. It is reassuring that the hardness increased by 10.5% (33.6 ± 0.0.9 MPa) when VE appeared in accelerated aging process. The improvement of this property can be attributed to the antioxidant effect of VE, which slows down the oxidative degradation rate of the nanocomposites. Therefore, in the process of accelerated aging, VE can improve the hardness when compared with the nanocomposite without soaked in VE solution.

### 3.6 Biotribological property

**Fig. 7:**
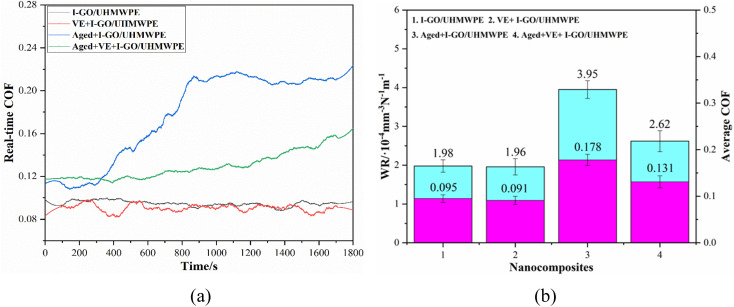
Real-time friction coefficient (a), average friction coefficient and wear rate (b).

The variation of COF with real-time in calf serum lubricating medium was shown as Fig. 7(a). It can be seen that in the whole friction process, the COF of the nanocomposites soaked in VE decreases slightly, but changed little. The COF of the nanocomposites without accelerated aging is basically in a stable state without obvious fluctuation. On the contrary, after accelerated aging, the COF changed greatly and obvious fluctuations can be seen, and many obvious fluctuation peaks on the COF curves. The COF of the nanocomposite soaked in VE was reduced to a certain extent, compared with that not soaked in VE solution. At the stage of 0–900 s, the COF of the nanocomposite after accelerated aging has been in a rapid growth stage. After 900 s, the increase of the COF is relatively slow, and not stable until the end of the experiment. Similarly, though the COF of the nanocomposite soaked in VE solution first and then accelerated aging treatment has been increasing slowly, it still remains unstable.

Average friction coefficient and wear rate of the nanocomposites was shown in Fig. 7(b). The results indicate that the variation trends of average COF and WR are nearly the same, that is, large COF corresponds to high WR. The COF of I-GO/UHMWPE nanocomposite is 0.095 ± 0.008 and decreased by 4.2% after VE immersion (0.091 ± 0.009), this is mainly because the oily VE acts as a lubricant in the tribological process and plays a role in reducing the COF. In addition, the free radicals produced during irradiation may react with O_2_ to form oxides, which increases the wettability of the nanocomposites. The oxygen-containing functional groups on GO surface also increased the wettability of the samples to a certain extent. Besides, the oxides may react with the protein in calf serum solution and formed a lubricating film on the surface.^27^ The above factors work together to reduce the average COF. After accelerated aging, the average COF of the nanocomposite was 0.178 ± 0.012, which increased by 87.4% compared with that non-aged. In the process of accelerated aging, the sample was in high temperature air for a long time, the movement speed of free radicals generated by irradiation increases, and a large number of free radicals reacted with oxygen to form oxides, which reduces the mechanical properties of the sample, such as hardness, so increased the average COF. In addition, in the process of tribological process, the oxides on the surface is easy to peel off from the matrix by external force, those abrasive particles increased wear, making the COF fluctuate greatly and further raise. For the nanocomposite soaked in VE solution first and then accelerated aging, the average COF is 0.131 ± 0.013, which is 26.4% lower than that without VE solution. Although in the process of accelerated aging, VE will further diffuse to the interior of the nanocomposite under the action of high temperature, and its lubrication will be weakened. On the whole, the COF of the nanocomposite is still reduced, which is mainly due to following two aspects. On the one hand, VE has good ability to absorb and stabilize free radicals, and can reduce the number of free radicals. On the other hand, in the process of accelerated aging, VE on the surface has the ability to slow down the diffusion of oxygen to the interior of the nanocomposite and reduce the formation of oxides, avoiding the reduction of mechanical properties to a certain extent, so the average COF decreases.

Meanwhile, WR is in the same situation. In calf serum, the wear rate of I-GO/UHMWPE was (1.98 ± 0.16) × 10^−4^ mm^3^ N^−1^ m^−1^, and decreased to (1.96 ± 0.21) × 10^−4^ mm^3^ N^−1^ m^−1^ after VE immersion, the slight decrease is mainly due to the lubrication effect of VE. After accelerated aging, the WR of the nanocomposites was (3.89 ± 0.23) × 10^−4^ mm^3^ N^−1^ m^−1^, which increased by 96.5% compared with the nanocomposite without accelerated aging. For those samples that have undergone VE diffusion first and then followed with accelerated aging treatment, the WR was (2.62 ± 0.27) × 10^−4^ mm^3^ N^−1^ m^−1^, which increased by 96.5%. Similar to the above analysis, accelerated aging causing oxidative degradation of the nanocomposite and decreased of the hardness. In addition, the oxides peeled off from the surface acts as wear particles, further increasing the WR. The WR of the nanocomposite soaked in VE solution decreased by 26.5% after accelerated aging, which is similar to the above analysis. On the one hand, VE on the surface can stabilize free radicals, hindering the diffusion of oxygen into the matrix and reducing oxidative degradation of the nanocomposite, thus avoid the reduction of mechanical properties to a certain extent. On the other hand, the lubrication effect of VE also reduces the WR. For UHMWPE and its composites, the thermal mechanical properties also affect the biotribological properties. Generally speaking, composites with better thermal properties also have better mechanical properties, which is beneficial for reducing weight loss during friction and wear processes, thereby reducing wear rates. In addition, materials with better thermal mechanical properties are also more wear-resistant and usually have better wear morphology.

**Fig. 8:**
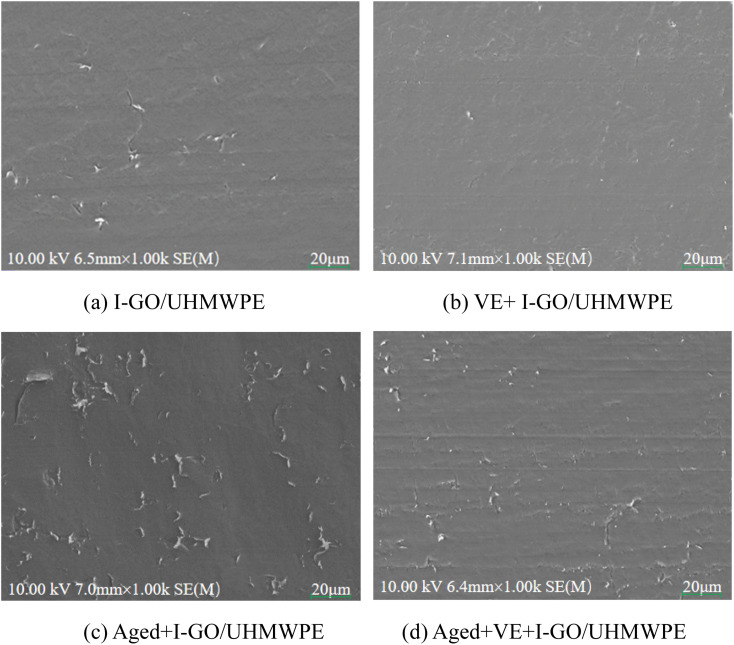
SEM images of worn surface: I-GO/UHMWPE (a); VE+I-GO/UHMWPE (b); Aged+I-GO/UHMWPE (c); Aged+VE+I-GO/UHMWPE (d).

SEM images of worn surface of the nanocomposite was shown in Fig. 8. Fatigue cracks appeared on the surface of I-GO/UHMWPE nanocomposite and it belongs to fatigue wear. After immersion in VE, the fatigue wear is significantly reduced, the worn surface was accompanied by fewer, shallower depth and shorter length cracks, which indicates that VE protects the nanocomposite from damage. Obviously, accelerated aging leading to serious fatigue wear and abrasive wear. In the process of accelerated aging, free radicals reacted with oxygen to form oxides, the molecular chain broken and the length became shorter, resulting in reduced mechanical and biotribological properties.^36^ In addition, the abrasive particles melted into calf serum to form three-body abrasion, which aggravates the deterioration of worn morphology. Therefore, a large number of pits appeared on the worn surface, resulting in an increase of surface roughness and COF. In the presence of VE, the worn surface of accelerated aging nanocomposite was significantly improved. VE acts as a lubricant to reduce wear, as analyzed above. In addition, the presence of VE also reduces the formation of surface oxides and avoids three-body abrasion.

### 3.7 Anti-friction and wear resistance mechanism

**Fig. 9:**
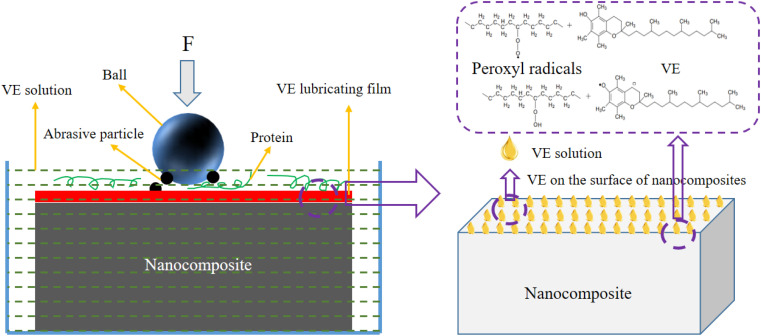
Anti-friction and wear resistance mechanism of VE.

Anti-friction and wear resistance mechanism of VE was shown in Fig. 9. Calf serum contains a large amount of protein, and the functional groups on the protein may grafted into the nanocomposites to lubricate the wear process. After accelerated aging treatment, a large number of abrasive particles were produced, the abrasive particles, grinding ball and nanocomposite form three-body abrasion, which intensifies the increase of COF and WR. VE, an oily substance, can act as a lubricating film to reduce wear. In addition, VE also has the function of stabilizing and absorbing free radicals (peroxyl radicals), which can reduce the formation of oxides and reduce the degree of three-body abrasion. For the improvement of biotribological properties of accelerated aging nanocomposites, indeed, VE is an excellent anti-friction and wear resistant material. In addition, it can be found that the presence of VE during accelerated aging can indeed slow down the decrease in the performance of nanocomposites to a certain extent.

## 4. Conclusions

The main purpose of this work is to investigate the effect of accelerated aging on the performance of irradiation cross-linked GO/UHMWPE nanocomposite after VE diffusion, the following conclusions can be concluded.

(1) VE diffusion hardly affects the melting point, crystallinity, thermal stability and gel content of the nanocomposite, as well as hardness. However, the oxidation resistance increased by 21.8%, and the friction coefficient and wear rate decreased slightly. (2) Accelerated aging increased the melting point and crystallinity of the nanocomposite, but the thermal stability and gel content decreased. In addition, the oxidation index increased by 60.2%, resulting in the decrease of hardness by 18.1%, and the increase of friction coefficient and wear rate by 99.5% and 87.4%, respectively. (3) In the process of accelerated aging, the presence of VE can significantly improve the above properties of the nanocomposite. In particular, the oxidation index decreased by 21.1%, and the friction coefficient and wear rate decreased by 33.7% and 26.4%. (4) After accelerated aging, the wear forms of the nanocomposite are fatigue wear and abrasive wear, while VE can play the role of anti-friction and wear resistance.

However, there are still some limitations in this study. For example, tensile performance, small punch tests and so on. In addition, further research is needed on the morphology of wear particles of nanocomposites and the biotribological properties under the conditions of pin-on-disc (POD) test.

## Data availability

All relevant data are within the manuscript and its additional figure files.

## Conflicts of interest

There are no conflicts to declare.
